# Dentition

**Published:** 1861-03

**Authors:** C. S. Chittenden

**Affiliations:** Hamilton, C. W.


### DENTITION.

Read before the Michigan Dental Association,

BY C. S. CHITTENDEN, HAMILTON, C. W.

SECOND dentition was the subject on which I was to write; but as second dentition is so intimately connected with *first* dentition, the first being incomplete when the second commences, and both being carried on at the same time and by nearly the same process, it renders it exceedingly difficult for me to speak of the second without also speaking of the first. I propose, therefore, to speak of *dentition*, instead of second dentition. We *know* something about the matter,—we think we know a good deal more. We know that the teeth exist, that they possess a certain form, and that they are composed of enamel, cementum and ivory, or tooth-bone, and that they contain a nerve, an artery, and a vein, and that they are used for masticating the food, and that they greatly assist articulation. We know that their substance is made up of the salts of lime, soda, etc.; but we do not know the modus operandi of their eruption, any better than we know, as the old song says:

"How oats, peas, beans and barley grow."

Nature seems to know what is best for us, and to attend to our wants without our asking. When the child is first ushered into being, we find the mouth exactly fitted for the kind of nourishment which nature has provided for it. Its delicate stomach requires no solid food, nor could it digest it were it fed on such, until nature has prepared its stomach for it; but so soon as the stomach is prepared to digest solid food, we find kind nature attending to its wants, by sending out the teeth for the purpose of masticating it, thus allowing each particle to become completely saturated with the saliva, before it is introduced into the stomach. Anatomists and physiologists do not agree at all as to the operation of nature in the production of the teeth. It is often asserted that the teeth of the second dentition are of a denser and harder substance than those of the first; and the reason given is, that the lactic fluid does not contain a sufficient quantity of bone making material, to form the teeth which are to last for life. But analysis proves that there is very little difference in the material of both sets. If it were true that the permanent teeth are denser and less liable to decay than the deciduous ones, because we eat solid food, we certainly ought to expect that the dentes sapientiæ would be the most perfect in the mouth, and would outlast all the others; when we know that of all the teeth they are the frailest and soonest lost. It appears to me we must look for some other solution of the matter. It is well known that when any portion of the human frame in a state of health, as the muscles for instance, are exercised for a length of time in a certain direction, that there is an increase in the size and strength of the parts, in consequence of the greater amount of the secretions which have been sent to them, and that atrophy, to a certain extent, succeeds a want of exercise. We all see such examples every day. Is it not quite probable that there may be a parallel drawn between the flow of the secretions, which go to increase the size of the muscles, and the flow of secretions which go to the sacs containing the embryo teeth?

Let us suppose a case. Let us take a healthy child at its third or fourth month. It has all the elements of growth, as its increase in size and its hardened bones give full proof. Its muscles are large, firm and strong, showing plainly that there has been no lack in the supply of the secretions which have been sent to them. The bones, too, by their size and firmness, show us that nature has been busy with them at the same time, depositing the salts of lime, magnesia, soda, etc., upon them; and so on throughout the whole system, increasing the size and strength of those parts which the child uses most. As it gets larger, its stomach begins to feel the necessity of being supplied with hardier food—food which must be saturated with the saliva before it can be digested, and the child seems constantly inclined to bite whatever it can get into its mouth.

Soon after this we begin to see indications of the growth of the teeth, showing that nature has directed those salts of which the teeth are composed, to be deposited little by little on them, until they make their appearance through the gums, and continues the process till the fangs are perfect. But do we know how this is done? Do we know any more about this than we do of the manner in which carbon enters into the formation of vegetable life?

We *think* we know that the bone material is taken from the food, and carried to the secretive organs and deposited in or on the bones. We know that some kinds of food contain a much greater quantity of bone material than others, and that it is a pretty well established fact that the teeth of those who for generations have lived almost exclusively on coarse food, are better, whiter and harder, and that they last much longer than the teeth of those who fare sumptuously every day.

Now, can we not form a pretty safe conclusion, from what we know, with regard to defective dentition and defective teeth in childhood?

For the last twelve years, my practice, to a great extent, has been among the Scotch, whose staple article of diet, at least for breakfast, is unbolted oat meal. I have found that the teeth of children brought up on this food are erupted with very little difficulty, are less liable to decay in childhood, and from the fact that they are retained in the mouth till the absorbents have carried the fangs away, the second set are more regular and more fully developed than the teeth of the natives of this country. Are they not indebted to a great extent for their fine teeth to their manner of living? Now, if such be the case, ought we not to use our endeavors to induce parents to give their children such food as will tend most to bring about the desired result? Shall we not have earned the heartiest thanks of the rising generation?

But to my mind there is another great source of mischief, which is not sufficiently regarded. I mean the practice of saturating the system with outlandish doses of mercurial medicines. We are all called daily to attend to the teeth of those who have been "murdered alive" with this drug. We know that mercury produces "great rottenness of the bones," and can we expect the offspring of parents whose whole systems are filled with this dreadful drug (I mean *dreadful*, because dreadfully used), to be healthy? and if not healthy, how can we look for any thing better in shape of teeth than the black, unsightly, disgusting apparatuses, which we see from day to day?

But as our specialty is more for the remedying of existing defects, than for preventing them, we must look for the best means for alleviating existing suffering. For this purpose, I have found one or two remedies sufficient for most cases. Of course, I would not advise the *mere dentist* to attempt to treat difficult cases; but, as we generally meet them, I think we may do so with perfect safety. For instance, we are often called upon to attend to children whose teeth are slow in coming through the gums; with relaxed bowels; great nervousness, etc.; scrofulous children; children who seem to have very little life force left in them. For such cases, I have found calcaria carbonica, in what Homeopaths call the third potency, given about once a day, for a short time, to be a most excellent remedy—a remedy which has done, is doing, and will do more good than any other with which I am acquainted. Lime water may often be used with good success in cases of slow dentition, by those people who are afraid of being benefited by Homeopathy.

The dentist should never attempt to assume the functions and duties of the physician, least of all, that big sounding word "Doctor;" but he may be able, from his study and experience, to give parents many useful hints.

With regard to the extraction of the deciduous teeth, it is difficult to conceive the mischief that is done by the perambulating doctors, in teaching parents to have the deciduous teeth extracted, to allow the permanent ones to come in regularly. It is often impossible for me to convince mothers that it will not be best to extract these teeth. The general impression seems to be that the neglecting to have the deciduous teeth extracted, *sometimes* before the permanent ones should make their appearance, is the sole cause of irregularity!

As I said before, nature seems to know what is best for us, and to attend to our wants without our asking; she will, if allowed to take her course, throw out the deciduous teeth at the proper time, in almost every instance.

My practice is never to extract a deciduous tooth unless there is an actual necessity for it, and I never consider that there is a necessity, unless the permanent tooth is about making its appearance, or there is disease, which renders the tooth too painful to be retained in the mouth.

### DENTAL FEES.

Essay read before the Michigan Dental Association.

